# The PD-1 Interactome

**DOI:** 10.1002/adbi.202100758

**Published:** 2021-06-25

**Authors:** Qi Wang, Kankana Bardhan, Vassiliki A. Boussiotis, Nikolaos Patsoukis

**Affiliations:** Department of Medicine Beth Israel Deaconess Medical Center Harvard Medical School, Boston, MA 02215, USA

**Keywords:** programmed death-1 interactome, programmed death-1 pathway, SHP-1, SHP-2, T cell activation

## Abstract

T cell activation is a fine-tuned process that involves T cell receptor and costimulation signals. To prevent undue activation of T cells, inhibitory molecules including PD-1 (programmed death 1) are induced and function as brakes for T cell signaling. In a steady state, the interaction of PD-1 with its ligands PD-L1 (B7-H1, CD274) and PD-L2 (B7-DC, CD273) maintains peripheral immune tolerance. However, the expression of PD-L1 on tumor cells and interaction with PD-1 on T cells dampen anti-tumor immunity. Therapeutic inhibitors of the PD-1 pathway have revolutionized tumor immunotherapy. Unfortunately, the majority of patients do not develop sustained anti-tumor responses. However, the knowledge about unique PD-1 interactions and their role in mediating PD-1 inhibitory signals is currently limited. Advances in the mechanistic understanding of the molecular and signaling integration of the PD-1 pathway could unleash the great potential in tumor immunotherapy by allowing the development of combinatorial approaches that target not only PD-1 and its ligands but also its unique downstream signal mediators. In this review, the current advances in understanding the mechanisms of extracellular and intracellular PD-1 interactions and their significance in potential future therapeutic approaches are discussed.

## 1. Introduction

Based on the classic two signal model of T cell activation,^[1]^ in order to be fully activated, T cells require two different signals, one signal derived from the T cell receptor (TCR) and a second signal derived from costimulatory receptors. First, the cognate antigen loaded on major histocompatibility complex (MHC) presented by antigen-presenting cells (APC) is recognized by the TCR, which initiates T cell activation. In parallel, stimulatory signals provided by interaction of CD28 on T cells and CD80 (also known as B7.1) or CD86 (also known B7.2) on APC further propel T cell activation. To counteract activation signals, negative regulators such as cytotoxic T-lymphocyte-associated protein (CTLA-4, also known as CD152) and programmed death 1 (PD-1) are upregulated. CTLA-4 competes with the positive costimulatory receptor CD28 for binding to CD80 and CD86 or by diminishing their expression through transendocytosis,^[2]^ whereas PD-1 binding with its ligands inhibits positive signals from TCR and CD28. These inhibitory receptors serve as immune checkpoints to regulate appropriate T cell activity and prevent the breaking of immune tolerance.^[3]^

PD-1 is expressed on activated T cells, B cells, natural killer (NK) cells, monocytes, dendritic (DC) cells, and myeloid progenitor cells.^[4]^ There are two ligands of PD-1, PD-L1 (also known as B7-H1, CD274) and PD-L2 (also known as B7-DC, CD273). PD-L1 is widely and constitutively expressed on both hematopoietic cells (including T cells, B cells, DC cells, and other myeloid cells) and non-hematopoietic cells (including vascular endothelial cells and pancreatic islets), while PD-L2 expression is more restricted to APC such as macrophages and DC cells.^[4]^ PD-L1 is also expressed on many tumor types, in which expression depends mainly on inflammatory cytokines, although some oncogenic mutations also contribute to PD-L1 expression.^[5]^ The expression of PD-L1 on tumor cells led to the hypothesis that PD-L1 greatly suppresses T cell killing of tumor cells through PD-1/PD-L1 interaction. Indeed, antibody-mediated blockade of the PD-1/PD-L1 axis unleashes T cell-mediated tumor killing with remarkable, although limited, effects in the clinic.^[6]^

PD-1 consists of an Ig-like binding domain, a transmembrane domain, and an intracellular tail, which contains an immunoreceptor tyrosine-based inhibition motif (ITIM) and an immunoreceptor tyrosine-based switch motif (ITSM).^[7]^ Upon binding to its ligands PD-L1 or PD-L2, the intracellular tyrosines of PD-1 rapidly become phosphorylated and recruit the tyrosine phosphatases SH2 domain-containing protein tyrosine phosphatase SHP-2 (PTPN11) and the homologous SHP-1 (PTPN6), which lead to dephosphorylation of both TCR and other costimulatory signaling components.^[8–11]^ This event ultimately results in attenuation of T cell activation, proliferation, and cytokine production.^[4]^ In this review, we will discuss recent findings on extracellular and intracellular mechanisms of PD-1 interaction modes and how they shape T cell activation. Better understanding of the molecular interactions within the PD-1 pathway will pave the way for new therapeutic interventions to improve tumor immunotherapy.

## 2. Expression and Cell-Specific Function of PD-1

PD-1 is not expressed on naïve T cells but it is rapidly upregulated on activated T cells and B cells.^[12]^ PD-1 expression on early activated T cells and effector T cells (Teff) inhibits immune responses to viral infections.^[13]^ During persistent antigen stimulation as in chronic infection and cancer, PD-1 is expressed on dysfunctional T cells, often termed exhausted T cells.^[14]^ PD-1 is also involved in regulatory cell (Treg) development since ligation of PD-L1 with PD-1 on naïve T cells can promote differentiation of induced Treg cells.^[15]^ Recently, it was shown that PD-1 expression on Treg cells versus CD8^+^ Teff cells could be used as a biomarker to predict efficiency of anti-PD-1 immunotherapy in several cancers.^[16]^ PD-1 blockade on PD-1^hi^ Treg cells enhanced their suppressive function and led to tumor progression.^[16,17]^ Although PD-1 was found highly expressed on exhausted T cells after chronic infection, deleting PD-1 not only did not improve but further impaired and delayed CD8^+^ memory T cell responses during chronic viral infection.^[18]^ In the germinal center, both T follicular cells (Tfh) and B cells express PD-1 and interaction of PD-1 on T cells with PD-L1 on B cells is essential for B cell optimal class switching and affinity maturation.^[19]^ Notably, a later study found that PD-1 signaling also affects the migration and function of Tfh cells in the germinal center, involving inhibition of PI3K downstream of CXCR5 signaling and downregulation of CXCR3.^[20]^

PD-1 is also expressed on various innate immune cells. PD-1 is induced by toll-like receptor signaling on mouse splenic DCs and negatively regulates function of DC cells during Listeria infection.^[21]^ In ovarian cancer patients, PD-1 was co-expressed with PD-L1 on tumor-infiltrating DC (TIDC) cells and blunted TIDC function by inactivating the NF-kb pathway.^[22]^ Expression of PD-1 on NK cells limits their function and PD-1 blockade restores NK cell anti-tumor function.^[23]^ ILC2 cells from adipose tissue of obese mice also upregulate PD-1 in a manner that depends on TNF and IL-33. PD-1 expression on ILC2 cells correlated with impaired function by recruiting PD-L1^hi^ M1 macrophages.^[24]^ Notably, both human and mouse tumor-associated macrophages (TAM) express PD-1, which negatively correlates with phagocytic activity against tumor cells.^[25]^ Recently, a previously unappreciated role of PD-1 on myeloid cell-dependent effectiveness of tumor immunotherapy was demonstrated.^[26]^ During tumor progression, myeloid progenitor cells constantly differentiate into multiple subsets-in a process known as emergency myelopoiesis. PD-1 was gradually induced on all myeloid cell subsets in several mouse tumor models by the emergency-myelopoiesis cytokine granulocyte colony-stimulating factor. Conditional deletion of PD-1 on myeloid cells prevented accumulation of myeloid-derived suppressor cells, while promoting a shift toward inflammatory myeloid cells and, unexpectedly, led to better control of tumor growth than T cell-specific PD-1 deletion. This could be explained by alterations to the metabolic program of myeloid cells after PD-1 ablation, including increased intermediates of glycolysis, the pentose phosphate pathway, and the Krebs cycle. Most prominently, the accumulation of cholesterol in PD-1-deficient tumor-bearing mice rewired function of macrophages and DC cells, promoted tumor antigen presentation, activation of T cells, and memory formation. These results highlight the importance of myeloid cell-intrinsic PD-1 in regulating tumor driven myeloid cell fate, function, and metabolism and indicate that this pathway might be a key mechanism of successful immune checkpoint blockade.^[26]^

## 3. Interaction of PD-1 with Its Ligands

**Figure 1.**
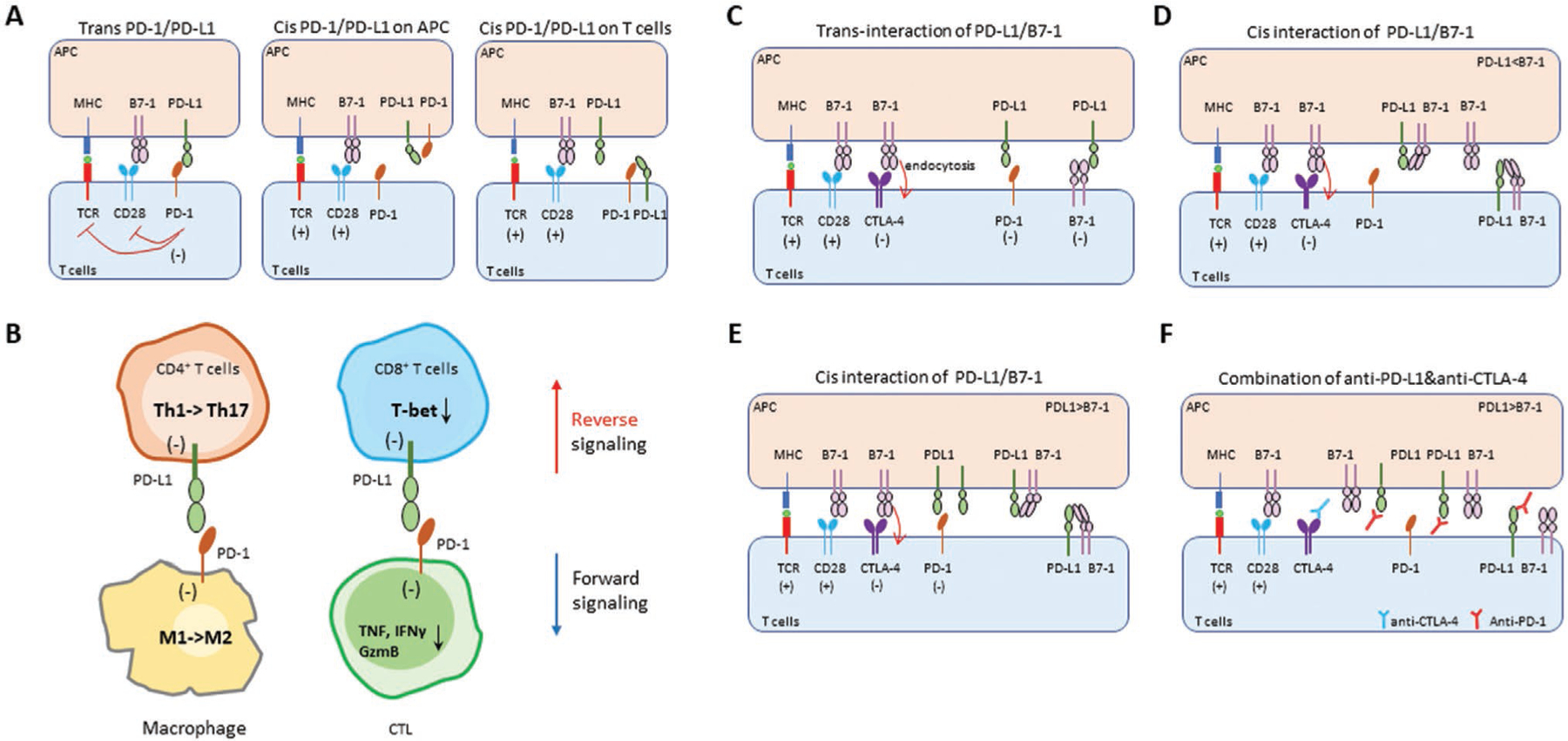
Interaction network of PD-1 with its ligands. A) The classic *trans* interaction of PD-1 on T cells and PD-L1 on APC reduces T cells activation via inhibition of TCR and CD28 signaling (left panel). PD-1 is also expressed on innate immune cells, in which the *cis* interaction of PD-1/PD-L1 might result in less free PD-L1 to bind PD-1 on T cells, thus prevent PD-1 inhibitory signaling on T cells (middle panel). As PD-L1 is also expressed on T cells and is further upregulated after T cell activation, the *cis* interaction of PD-1/PD-L1 might take place on T cells after T cell activation (right panel). This *cis* interaction of PD-1/PD-L1 and self-inhibition on T cells might prevent a continuous activation state. B) PD-L1 is highly expressed on T cells, thus the bidirectional signal of PD-L1 may change the functional fate of T cells in the tumor microenvironment. In this setting, PD-1 might act as a ligand for PD-L1 and transmit an inhibitory signal to T cells and macrophages in the tumor tissue. The backward singling of PD-L1 might shift CD4^+^ T cells from Th1 to Th17 and limit cytotoxic effects of CD8^+^ T cells in the tumor. The forward signaling of PD-L1 changes inflammatory macrophages (M1) to pro-tumor (M2) macrophages. In addition, forward signaling of PD-L1 via PD-1 can reduce production of inflammatory cytokines and effector cytotoxic T lymphocytes (CTL). Overall, the bidirectionality of PD-L1 signaling dampens immune responses against tumor. GzmB, Granzyme B. C) T cell activation requires a first signal from the TCR and second co-stimulatory signal from CD28 (CD28/B7–1). To counteract this process, inhibitory signals from CTLA-4 (CTLA-4/B7–1) and PD-1 (PD-1/PD-L1) act as brakes for further activation of T cells. CTLA-4 mediates its inhibitory function via endocytosis after binding with its ligand B7–1. The *trans* interaction of PD-L1 and B7–1 was previously reported to inhibit T cell activation. D) The *cis* interaction of PD-L1 and B7–1 on both APC and T cells. In co-culture of T cell and APC, the *cis* interaction of PD-L1 and B7–1 blocks the *trans* interaction of PD-L1/PD-1, thus making PD-L1 less available on APC for binding PD-1 on T cells, resulting in improved T cell activation. However, this relies on more expression of B7–1 than PD-L1 on APC cells. Interestingly, the *cis* interaction of PD-L1/B7–1 has no effects on the *trans* B7–1 binding to CTLA-4 and CD28. E) When PD-L1 outnumbers B7–1 on APC cells, the *cis* interaction of PD-L1 and B7–1 might have limited effects on *trans* interaction of PD-L1/PD-1. The excess of PD-L1 on APC could still bind to PD-1 and transmit inhibitory signals to T cells. F) When PD-L1 outnumbers B7–1 on APC cells, blocking *cis* interaction of PD-L1/B7–1 and *trans* interaction of PD-L1/PD-1 by anti-PD-L1 can further activate T cells. However, in this case, the released B7–1 on APC might bind to CTLA-4 on T cells and cause their inhibition. Thus, combination of anti-PD-L1 and anti-CTLA-4 under these conditions could maximize T cell activation and may be an efficient approach to improve tumor immunotherapy.

The classic PD-1 pathway involves the *trans* interaction between PD-1 on T cells and PD-L1 on APC (or tumor cells), which leads to attenuation of T cell response (Figure 1A). A recent study found that *cis* interaction also takes place between PD-1 and PD-L1 on the same surface of tumor cells or APCs.^[27]^ By using protein-reconstituted membrane lipid bilayers and cell-based assays, the study demonstrated that *cis* interaction of PD-1 and PD-L1 on tumor cells or APC impedes the *trans* interaction of PD-L1 on tumor or APC cells with PD-1 on T cell, and thus represses the canonical PD-L1/PD-1 inhibitory signaling. In this setting, *cis* interaction of PD-1 and PD-L1 on tumor cells or APC rendered T cells more activated.^[27]^ However, the possibility of *cis* interaction between T cell intrinsic PD-1 and T cell intrinsic PD-L1 cannot be excluded (Figure 1A), since both PD-1 and PD-L1 are expressed on activated T cells.^[28]^ Besides, when PD-1 expression level is lower than PD-L1 on tumor cells or APC, the residual PD-L1 could still mediate suppressive function via binding PD-1 on T cells *in trans*. This latter scenario may be more physiological since PD-1 expression is lower than PD-L1 on tumor cells or APC.^[28]^ Further studies are required to validate these possibilities (Figure 1A).

Growing evidence suggests that ligation of PD-L1 could transmit reverse signaling to T cells or tumor cells.^[29,30]^ It was recently reported that in a pancreatic tumor model, tumor-infiltrating T cells expressed high levels of PD-L1, which could provide bi-directional signaling via PD-1/PD-L1 axis.^[30]^ In this setting, since most activated T cells already express PD-1, it is not clear whether PD-L1 expressed by the same T cells would function as a ligand or a receptor (Figure 1B). The study showed that PD-L1 expressed by T cells transduced bi-directional signals that converged to suppress a network of immune cells in the pancreatic tumors (Figure 1B). In the reverse signaling mode, PD-L1 functions as a receptor to transmit reverse signals back into T cells once PD-L1 binds PD-1 on neighbor cell (T cells or macrophages, *trans* interaction of PD-L1/PD-1). Reverse signals from PD-L1 to T cells limited Th1 polarization of CD4^+^ T cells while promoted conversion of CD4^+^ T cells to Th17. In addition, reverse signals from PD-L1 prevented CD8^+^ T cells from gaining cytotoxic effector functions. In the forward signaling mode, PD-L1 functions as a ligand to engage PD-1 expressed on T cells or macrophages, resulting in tolerant T cells and M1 to M2 conversion of macrophages. In this regard, PD-L1 expressed by T cells provides a new mechanism of T cell-T cell and T cell-macrophage crosstalk via PD-1 and leads to a spectrum of suppressive signals in pancreatic tumors (Figure 1B). Although PD-L1 might transmit reverse signals to T cells, the involved signaling pathway remains unknown. Besides, it is not clear whether CD80 (B7–1), which can also bind PD-L1, would similarly mediate reverse signaling through PD-L1. Last, whether these bi-directional signals of PD-L1 occur on the same T cell surface (*cis* interaction of PD-1/PD-L1) still needs to be determined.

The PD-L1/B7–1 *trans* interaction between APC and T cells was initially reported using cell-protein interaction assays and surface plasmon resonance (Figure 1C).^[31]^ However, later studies demonstrated that *cis interaction* existed between PD-L1 and B7–1 on the same cell surface of APC or tumor cells^[32]^ and that this *cis* interaction relies on flexible, orientation-permissive PD-L1 on APC.^[33]^ Another study further verified the *cis* interaction of PD-L1/B7–1 on the same surface of APC by using various cell co-culture, biochemistry assays, and genetic knock-in mice (Figure 1D). The study demonstrated that *cis* PD-L1/B7–1 interactions disrupt the *trans* PD-1/PD-L1 interactions, resulting in impaired PD-1-mediated T cell suppression.^[34]^ Remarkably, the *cis*-PD-L1/B7–1 interactions do not interfere with B7–1/CD28 or B7–1/CTLA-4 binding.^[34]^ In parallel, it was reported that *cis*-PD-L1/B7–1 interaction on APC restricts PD-L1 binding to PD-1 *in trans*, while B7–1/CD28 binding is maintained. However, that study found that *cis* interaction of PD-L1/B7–1 disrupts the B7–1/CTLA-4 binding, resulting in reduced CTLA-4-mediated transendocytosis of B7–1 and inhibitory signaling of CTLA-4.^[35]^ Based on these findings, when PD-L1 molecules outnumber those of B7–1, despite *cis* interaction of PD-L1/B7–1, the residual PD-L1 could still transmit inhibitory signals to T cells (Figure 1E). In this setting, anti-PD-L1 could disrupt *cis* interaction of PD-L1/B7–1 and *trans* interaction of PD-1/PD-L1, however the released B7–1 might bind to both CTLA-4 and CD28. Further blockade of CTLA-4/B7–1 results in more activated T cell signaling via B7–1/CD28 interaction (Figure 1F). These findings provide evidence for a functional crosstalk between the PD-1 and CTLA-4 inhibitory receptors that depend on the abundance of PD-L1 and B7–1 expression on cancer cells or APC, and support the rationale for combining blockade of PD-L1 and CTLA-4 to improve clinical outcomes of tumor immunotherapy.

## 4. PD-1 Signaling and Mechanisms

**Figure 2.**
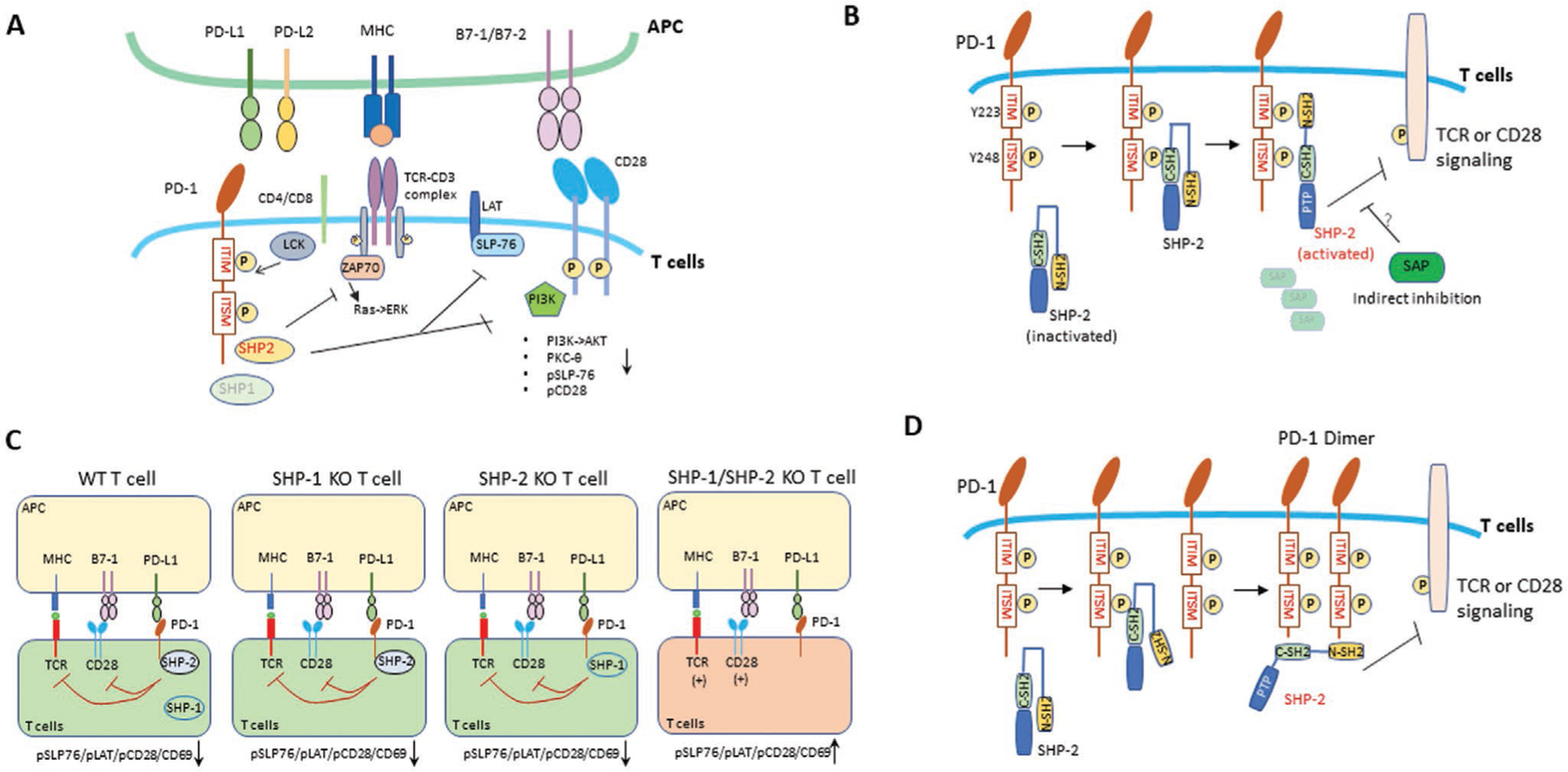
PD-1 signaling and interactors. A) T cell activation requires first signal from the TCR by engagement with peptide-loaded MHC. The recruited Src-family kinases, Lck and Fyn by TCR signaling rapidly phosphorylate ITIM and ITSM tyrosines of the cytoplasmic tail of PD-1 (pPD-1). Both SHP-2 and SHP-1 could be recruited to pPD-1, and promote downstream inhibitory signaling. These phosphatases can counteract positive signaling from TCR and CD28, which includes inhibition of ZAP70, SLP-76, protein kinase C (PKC-*θ*), phosphatidylinositol-3-kinase (PI3K), and the Ras signaling pathway. B) The self-inhibitory structure of SHP-2 binds to pITIM and pTISM of PD-1. SHP-2 contains two SH2 domains, N-terminal (N-SH2) and C-terminal (C-SH2), followed by a PTP domain. After C-SH2 binds to pITSM domain of PD-1, N-SH2 is released from the autoinhibitory conformation and binds to pITIM tyrosine of PD-1. By using a synthetic peptide (GST-PD-1 cytoplasmic tail) as bait to affinity purify candidate proteins in activated human T cells, signaling lymphocytic activation molecule-associated protein (SAP) was discovered in the PD-1 interactome. SAP blocked PD-1 inhibitory functions in T cells through indirect inhibition of SHP-2 activity. SAP did not show direct association with SHP-2 and might interact through a still unknown adaptor protein to inhibit PD-1 signaling. C) SHP-2 outcompetes SHP-1 for binding to phosphorylated PD-1 under physiologic conditions and inhibits both TCR and CD28 signaling. In the absence of SHP-1, SHP-2 could still inhibit T cell activation by downregulation of pSLP76, pLAT, pCD28, and expression of CD69. Interestingly, SHP-1 could mediate PD-1 inhibitory signaling in the absence of SHP-2. Double deletion of SHP-1 and SHP-2 on T cells showed complete loss of PD-1-mediated inhibition of CD28 and TCR signaling. D) Since pITSM of PD-1 could bind to N-SH2 domain of SHP-2, SHP-2 could bridge two pITSM motifs of PD-1 molecules through its N-SH2 and C-SH2 domains, which leads to dimerization of PD-1. In this model, the increased PTP activity of SHP-2 may inhibit TCR or/and CD28 signaling.

Most of our understanding of PD-1 signaling comes from studies of activated T cells (Figure 2A).^[36]^ PD-1 contains two tyrosine motifs in its cytoplasmic tail, an ITIM, and an ITSM, which become phosphorylated upon PD-1 engagement.^[36,37]^ Src-family kinases such as Lck/Fyn in T cells and Lyn in B cells are thought to be the main mediators of PD-1 phosphorylation.^[9,37]^ In T cells, phosphorylation of these two tyrosine residues leads to the recruitment of SHP-2 and SHP-1. Both SHP-2 and SHP-1 are composed of a central catalytic domain (protein tyrosine phosphatase, PTP domain) and two tandem SH2 domains at their N-terminus, N-SH2, and C-SH2.^[38]^ Mutagenesis studies showed that ITSM is the primary tyrosine related with recruitment of SHP-2 and inhibition of T cell activation.^[11,37,39]^ Upon activation, PD-1 engagement targets TCR downstream signaling, reducing phosphorylation of CD3*ζ*, zeta-associated protein of 70 kD (Zap70) and protein kinase C *θ* (PKC-*θ*).^[9]^ It has also been demonstrated that PD-1 blocks CD28-mediated activation of phosphatidylinositol-3-kinase (PI3K), phospholipase C*γ*2 (PLC*γ*2), and of serine-threonine kinase Akt by recruiting mainly SHP-2 and possibly SHP-1 (Figure 2A).^[11]^ The final outcomes of PD-1 signaling are reduced T cell activation, proliferation, cytokine production, and metabolic reprogramming.^[40]^ Notably, although it was initially thought that PD-1-mediated inhibition can be reversed by addition of IL-2,^[41]^ later studies provided evidence that IL-2 only partially restores PD-1-mediated signaling inhibition and does not repair blockade of cell cycle progression.^[42]^

Currently, it is still a debate which is the primary target of PD-1 (Figure 2A). It is widely accepted that PD-1 suppresses TCR signaling.^[43]^ However, CD28 has also been proposed as the primary target of PD-1.^[8]^ In parallel, another study showed that CD28 signaling on exhausted T cells in mice is required for anti-PD-L1 therapies to eliminate cancer cells and viral infections efficiently,^[44]^ but how PD-1 specifically modulates CD28 versus TCR signaling remains to be determined. Recently, a quantitative interactomic study demonstrated that both TCR and CD28 signals were inhibited by PD-1 (Figure 2C).^[45]^ Many T cell subsets (Treg, memory T cells, and exhausted T cells) express high levels of PD-1, but it is unclear whether PD-1 might specifically regulate TCR or CD28 signals in different T cell subsets.

As mentioned above, the PD-1 cytoplasmic tail contains two tyrosine motifs, ITIM (Y223) and ITSM (Y248) with ITSM being indispensable for PD-1 inhibitory function.^[10,11,39]^ Structurally, SHP-2 contains two SH2 domains, an N-terminal (N-SH2) and a C-terminal (C-SH2) SH2 domain followed by a PTP domain with N-SH2 binding to PTP in a closed autoinhibitory conformation. After C-SH2 binds to phosphorylated ITSM (pITSM) motif of PD-1, N-SH2 releases PTP, allowing PTP activity, and binds to phosphorylated ITIM (pITIM) of PD-1 (Figure 2B,D) in a two-step binding process.^[46]^ Recently, a study reported that both N-SH2 and C-SH2 domains of SHP-2 can bind to pITSM of PD-1. By employing mutational and biophysical approaches, this study showed that only Y248 is required for SHP-2 docking (Figure 2D). Based on this model, SHP-2 can bridge two pITSM tyrosines from two PD-1 molecules, forming a PD-1 dimer, which induces strong PTP activity.^[39]^ Importantly, this study showed that phosphorylation of PD-1 cytoplasmic tail also has an active role in PD-1 oligomerization mediated by PD-L1 because PD-1: PD-1 dimer formation induced by dimeric PD-L1 was impaired when phosphorylation of PD-1 cytoplasmic tail was abrogated by the expression of a kinase inactive Fyn. Thus, the stabilization provided by PD-1 ligands likely allows the appropriate proximity of two phosphorylated PD-1 molecules to induce binding of both SH2 domains and activation of SHP-2. Further studies are required to visualize this dimer of PD-1 brought together by SHP-2 in the immune synapse.

## 5. Intracellular Partners of PD-1

Although the extracellular interactions of PD-1 with its ligands (PD-L1 and PD-L2) are extensively studied, the intracellular interacting partners of PD-1 besides SHP-2 or SHP-1 phosphatases, remain a puzzle. By using affinity purification coupled with mass spectrometry (AP-MS), signaling lymphocytic activation molecule-associated protein (SAP) was found as candidate PD-1-interaction partner and was functionally and mechanistically examined for its contribution to PD-1 inhibitory responses (Figure 2B). In this report, SAP was highly enriched when using GST-PD-1 peptide as a bait to affinity purify interacting proteins from lysates of Jurkat T cells or primary T cells. T cells lacking functional SAP were hyper-responsive to PD-1 signaling, supporting its inhibitory role downstream of PD-1 (Figure 2B). Notably, SAP did not compete with SHP-2 for binding with PD-1, while opposed PD-1 function, potentially by acting as a molecular shield of key tyrosine residues that are targets for the tyrosine phosphatase SHP-2.^[47]^ Similarly, by using a synthetic peptide of PD-1 intracellular tail as bait, another study identified FBXO38 (F-Box Protein 38) as an interacting partner of PD-1 that mediates Lys48-linked poly-ubiquitination and subsequent proteasome PD-1 degradation.^[48]^

SHP-2 is the main PD-1 interaction partner inhibiting TCR signaling indirectly, whereas SHP-1 acts as a direct negative regulator of TCR signaling via its recruitment by ZAP-70, Lck, or THEMIS.^[49]^ A recent study showed that PD-1 blockade could still enhance tumor control in T cell-specific SHP-2 knockout mice, suggesting the possible existence of additional molecules involved in the PD-1 pathway.^[50]^ To identify PD-1-interacting partners in a more physiologic model, mice expressing a twin-strep-tag (OST) for affinity purification at the C terminus of endogenous PD-1 (PD-1^OST^ mice) were developed.^[45]^ By using T cells from these mice and PD-1^OST^-tag-engineered Jurkat cells in cell-based assays co-cultured with Raji cells, the AP-MS study quantitatively defined the PD-1 signalosome during T cell activation. The study also showed that SHP-1 could mediate PD-1 co-inhibitory function in the absence of SHP-2 on primary T cells and cell lines.^[45]^ In the absence of SHP-2, SHP-1 could still mediate PD-1 signals resulting in reduced level of tyrosine phosphorylation of CD28, LAT, SLP76, and ERK (Figure 2C).^[45]^ These results can explain the paradoxical finding that SHP-2 is dispensable for PD-1 signaling in vivo.^[50]^ This could also be explained by the involvement of a different phosphatase in SHP-2 deficient T cells. Intriguingly, an independent study reported that in SHP-1/2 double-deficient primary T cells, PD-1 could still potently inhibit cell proliferation and cytokine production, albeit more transiently than in wild type T cells, suggesting that PD-1 could suppress T cell signaling partially through yet unidentified mechanisms independent of SHP-1 and SHP-2.^[51]^ Although SHP-1 and SHP-2 were found as key components of the PD-1 signalosome, it is still not clear whether they might compete with each other to bind PD-1 under physiological conditions. The latter study reported that PD-1 selectively recruits SHP-2 over SHP-1, however, SHP-1 showed stronger phosphatase activity than SHP-2. Interestingly, B and T lymphocyte attenuator (BTLA) which, like PD-1, also contains two tyrosine domains (ITIM and ITSM), could preferentially recruit SHP-1 over SHP-2.^[51]^ These results might explain the functional differences of PD-1 and BTLA. Further work is needed to determine the largely unknown PD-1 interaction network and its role in mediated inhibitory mechanisms.

Recent phosphoproteomics studies and in silico predictions showed that PD-1 is a component of multimolecular complexes, where it might mount direct or indirect molecular interactions. By recruitment into such complexes, PD-1 might target multiple signaling substrates and affect distinct functions with important roles in T cell activation such as cytoskeleton reorganization, signal initiation, gene expression, and protein translation.^[52,53]^ Further studies are required to determine the biological relevance and implications of these findings in the context of disease models and clinical conditions.

## 6. Therapeutic Targeting of the PD-1 Interactome

The mandatory requirement of PD-1: SHP-2 interaction for induction of PD-1 inhibitory signaling and inhibition of T cell responses can also be a target of therapeutic intervention to improve the outcome of checkpoint immunotherapy. Recently, Fernandes et al, made the interesting observation that although immune checkpoint blockade therapy against PD-1 efficiently blocks the ligand activated signaling, it fails to inhibit a sustained, tonic, intracellular PD-1 signaling that continues to mediate T cell suppression.^[54]^ To overcome this inhibitory signaling, they designed a hetero-bispecific molecule that could recruit CD45 (a constitutively active cell surface tyrosine phosphatase expressed on lymphoid cells) to close proximity of PD-1 by binding to the extracellular domains of both molecules on T cell surface. As a consequence of this *in cis* interaction, the intracellular phosphatase domain of CD45 could mediate its enzymatic activity and dephosphorylate the phosphorylated tyrosine residues of PD-1 cytoplasmic tail, resulting in inhibition of both tonic and ligand-mediated PD-1 signaling. This PD-1 receptor inhibition approach by phosphatase recruitment (RIPR) showed superiority over checkpoint blocking antibodies, could potentiate T cell activation in vitro, and significantly reduced tumor burden in vivo in murine models of small cell lung cancer and colon adenocarcinoma. These observations suggest that targeting the phosphorylation-dependent PD-1: SHP-2 interaction, could be more efficacious than antibody-based checkpoint immunotherapy in cancer patients. It should be noted that tonic signaling by inhibitory receptors is important for immune homeostasis and its continuous existence mitigates autoimmunity. As a consequence, such therapeutic approaches might have more detrimental side effects and worse toxicity profile than antibody-based PD-1 immunotherapy.

Because PD-1 signaling targets multiple pathways, intense investigation is currently ongoing to determine whether combinatorial approaches might augment and enhance PD-1-mediated T cell activation and immune function, thereby extending the benefit of immunotherapy to patients who do not respond to PD-1 blockade alone. For example, simultaneous blockade of PD-1 and SHP-2 combined with radiotherapy provides superior anti-tumor effects in the context of tumors resistant to PD-1 immunotherapy alone.^[55]^ Activation of vaccinia related kinase 2 was recently shown to correlate with PD-1 signaling, and its pharmacologic inhibition combined with PD-1 blockade in in vivo syngeneic tumor models enhanced tumor clearance through T cell activation.^[56]^ In the same context, bispecific antibodies that deliver simultaneous blockade of PD-1 with other immune receptors, such as PD-L1^[57]^ or CD27,^[58]^ or growth factor receptors expressed in cancer cells such as Her2/Erb2,^[59]^ have shown promising results. In addition, small molecules that function as chemical inhibitors of PD-1: PD-L1 interaction are starting to emerge^[60,61]^ with the promise to overcome the limitations associated with the use of monoclonal antibodies including immunogenicity, sort half-life, and high cost. Such compounds not only diminish side effects but also have increased potency compared with monoclonal antibodies.^[60]^ These studies have paved the way for a new era of therapeutics in the field of checkpoint inhibitor blockade.

## 7. Concluding Remarks

The PD-1 pathway plays diverse roles in regulating immunity including cancer, infections, and autoimmune disease. Although our understanding of the PD-1 pathway has been translated into clinical immunotherapy that benefits some cancer patients, the majority of patients do not respond for long period of time and their cancer relapses and progresses. Combination of anti-PD1 therapy with other anti-tumor drugs or novel approaches of intervention to PD-1 interactome such as RIPR, holds promise for improving clinical outcomes. There are still many open questions in the clinical and basic research related with the PD-1 pathway. First, since all activated T cells express PD-1, how does the PD-1 pathway affect different T cell subsets such as effector, memory, regulatory and exhausted T cells? Second, since PD-1 is also expressed on several types of innate immune cells, when PD-1 blockade occurs on innate immune cells, what is the outcome of immune responses compared to PD-1 blockade on T cells? Last, since *cis* interactions of PD-1/PD-L1 axis and *cis* interactions of PD-L1/B7–1 were observed, it is paramount to investigate and distinguish the effects and functional outcomes of blocking the *cis* versus *trans* interactions of the PD-1/PD-L1 axis. Better understanding of the basic and interactome mechanisms of this pathway will allow the development of efficient combination therapies.
